# Pre-procedural IL-18, hs-CRP, and VEGF as predictors of adverse outcomes in acute myocardial infarction patients undergoing percutaneous coronary intervention

**DOI:** 10.3389/fmed.2026.1770624

**Published:** 2026-03-04

**Authors:** Jinlong Miao, Du Tao

**Affiliations:** Department of Cardiovascular Medicine, Lanzhou Second People's Hospital, Lanzhou, Gansu, China

**Keywords:** acute myocardial infarction, hs-CRP and VEGF, IL-18, percutaneous coronary intervention, prognosis

## Abstract

**Background:**

Identifying biomarkers that predict adverse outcomes after percutaneous coronary intervention (PCI) for acute myocardial infarction (AMI) could improve risk stratification and guide clinical management.

**Objectives:**

To evaluate the prognostic value of pre-procedural IL-18, hs-CRP, and VEGF levels in predicting major adverse cardiovascular events (MACEs) in AMI patients undergoing PCI. To assess their utility in risk stratification compared to traditional clinical parameters.

**Methods:**

This retrospective cohort study analyzed patients with AMI undergoing PCI between January 2023 and December 2024, with healthy controls for comparison. Serum interleukin-18 (IL-18), high-sensitivity C-reactive protein (hs-CRP), and vascular endothelial growth factor (VEGF) levels were measured before and after PCI. Patients were followed-up for 12 months to assess MACEs, including cardiac death, target vessel revascularization, ischemic stroke, and heart failure hospitalization. Patients were stratified into the good prognosis (no MACEs) and poor prognosis (MACEs) groups. Logistic regression and receiver operating characteristic (ROC) curve analyses were used to evaluate the prognostic value of these biomarkers.

**Results:**

A total of 170 AMI patients and 100 healthy controls were included. All three biomarkers were significantly elevated in AMI patients compared to controls and decreased after PCI (all *p* < 0.01). Pre-procedural IL-18, hs-CRP, and VEGF levels were significantly higher in the poor prognosis group (*n* = 40) than in the good prognosis group (*n* = 130) (all *p* < 0.01). Traditional clinical characteristics did not differ between outcome groups (all *p* > 0.05). Logistic regression identified all three biomarkers as independent predictors of poor prognosis (IL-18, OR = 26.075; hs-CRP, OR = 2.284; VEGF, OR = 1.643; all *p* < 0.001). ROC analysis demonstrated excellent discriminatory capacity, with area under the curve values of 0.803 for IL-18, 0.838 for hs-CRP, and 0.800 for VEGF (all *p* < 0.001).

**Conclusion:**

Elevated preprocedural IL-18, hs-CRP, and VEGF levels independently predict adverse outcomes in AMI patients undergoing PCI, offering superior risk stratification compared to traditional clinical parameters.

## Introduction

Acute myocardial infarction (AMI) is a cardiovascular emergency characterized by a sudden reduction or cessation of coronary blood flow, resulting in myocardial ischemia and necrosis (1). This condition represents a major public health challenge owing to its rapid onset, rapid progression, serious complications, and substantial impact on morbidity and mortality (2). The epidemiology of AMI in China has evolved considerably with economic development, showing a trend toward a younger age of onset and increasing incidence and mortality rates (3). Percutaneous coronary intervention (PCI) has become the cornerstone of AMI treatment, effectively restoring myocardial perfusion and preserving cardiac function (4). However, despite optimal revascularization and guideline-directed medical therapy, including antiplatelet and anticoagulant agents, a subset of patients continue to experience major adverse cardiovascular events (MACEs); defined as a composite of cardiac death, target vessel revascularization, ischemic stroke, and hospitalization for heart failure, following PCI, contributing to poor clinical outcomes (5). Early identification of biomarkers associated with post-PCI MACEs could facilitate risk stratification and guide targeted interventions to improve patient prognosis.

The inflammatory response plays a central role in cardiovascular complications by damaging the vascular endothelium, triggering inflammatory mediator release, inducing coronary vasospasm, activating coagulation pathways, and promoting plaque instability, ultimately precipitating MACEs (6). High-sensitivity C-reactive protein (hs-CRP) and interleukin-18 (IL-18) are key inflammatory mediators that amplify the inflammatory cascade, compromise endothelial integrity, and contribute to AMI pathogenesis (7, 8).

Several studies have investigated the prognostic value of inflammatory biomarkers in AMI patients undergoing PCI, though findings remain inconsistent and controversies persist. Regarding hs-CRP, Mincu et al. conducted a meta-analysis demonstrating that elevated preprocedural CRP levels predict cardiovascular risk following primary PCI in ST-elevation AMI (9). However, controversy exists regarding the optimal timing of measurement, threshold values for risk stratification, and whether CRP provides incremental prognostic value beyond established risk scores (10). For IL-18, Gao et al. reported that admission IL-18 levels predicted 60-day adverse events in ST-elevation AMI patients undergoing PCI (11), yet the evidence base remains limited compared to hs-CRP, and the clinical utility of IL-18 measurement in routine practice has not been established (12). Furthermore, most existing studies have evaluated these biomarkers in isolation rather than in combination, and direct comparisons of their predictive performance are scarce.

Vascular endothelial growth factor (VEGF) has demonstrated protective effects in experimental AMI models by improving myocardial function and reducing inflammation (13). Although VEGF expression remains low under physiological conditions, ischemia and hypoxia trigger substantial upregulation to promote endothelial cell proliferation and angiogenesis, potentially mitigating tissue hypoxia and improving clinical outcomes (14). Han et al. reported that serum VEGF levels predicted worse clinical outcomes in coronary heart disease patients after PCI (15), suggesting that markedly elevated VEGF may reflect the severity of underlying ischemic injury rather than a purely protective response. However, the prognostic role of VEGF in AMI specifically, and its comparative value against inflammatory markers, remains underexplored.

Current risk stratification approaches for post-PCI outcomes primarily rely on clinical risk scores such as the GRACE and TIMI scores, which incorporate traditional variables including age, hemodynamic parameters, and cardiac biomarkers such as troponin (16). While these models provide useful prognostic information, their discriminatory capacity is limited, and the integration of novel biomarkers reflecting inflammatory and angiogenic pathways may enhance risk prediction. Combined biomarker panels incorporating multiple pathophysiological pathways have shown promise in cardiovascular risk stratification (17), yet comprehensive evaluation of IL-18, hs-CRP, and VEGF together in AMI patients undergoing PCI has not been performed.

This study aimed to evaluate the relationship between preprocedural serum IL-18, hs-CRP, and VEGF levels and clinical outcomes in AMI patients undergoing PCI. Specifically, we sought to: (1) compare biomarker levels between AMI patients and healthy controls; (2) assess temporal changes in biomarker levels following PCI; (3) determine whether pre-procedural biomarker levels differ between patients with and without subsequent MACEs; (4) evaluate the independent prognostic value of each biomarker using multivariable regression analysis; and (5) compare the discriminatory performance of individual biomarkers for predicting adverse outcomes. By addressing these objectives, we aimed to provide evidence for the clinical utility of these biomarkers in risk stratification and to inform future biomarker-guided management strategies.

## Methods

### Study endpoints and outcome assessment

The primary endpoint was the occurrence of major adverse cardiovascular events (MACEs), defined as a composite of cardiac death, target vessel revascularization, ischemic stroke, and hospitalization for heart failure during 12 months of follow-up post-PCI. Secondary endpoints included individual components of the composite outcome. Patients were stratified into two groups based on clinical outcomes: those experiencing MACEs were classified into the poor prognosis group, whereas those who remained event-free constituted the good prognosis group. All reported events were adjudicated by an independent clinical event committee comprising two cardiologists who reviewed the source documentation.

### Study design and population

This retrospective cohort study was conducted at Lanzhou Second People’s Hospital to investigate the relationship between inflammatory and angiogenic biomarkers and clinical outcomes in patients with AMI undergoing PCI. The study protocol adhered to the principles of the Declaration of Helsinki and was approved by the Institutional Review Board of Lanzhou Second People’s Hospital. Written informed consent was obtained from all participants or their legal representatives.

**Figure 1:**
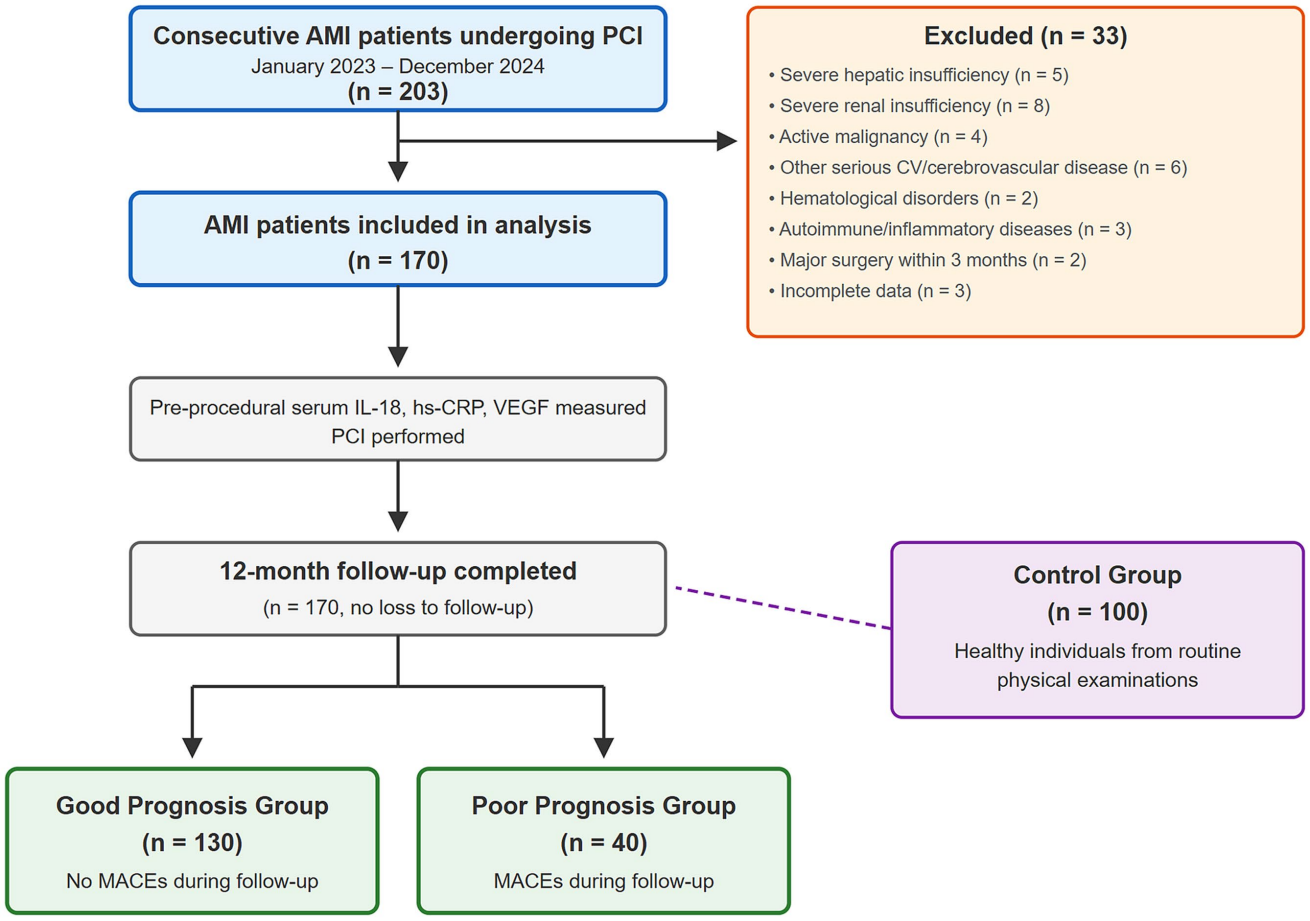
Patient enrollment flow diagram. AMI, acute myocardial infarction; PCI, percutaneous coronary intervention; CV, cardiovascular; MACES, major adverse cardiovascular events; IL-18, interleukin-18; hs-CRP, high-sensitivity C-reactive protein; VEGF, vascular endothelial growth factor.

We reviewed all consecutive patients diagnosed with AMI who underwent PCI at our institution between January 2023 and December 2024. Patients meeting inclusion criteria and without exclusion criteria were enrolled. For comparison, healthy individuals who underwent routine physical examination at our institution during the same period were recruited as controls; these individuals were frequency-matched to AMI patients by age and sex distribution and had no history of cardiovascular disease, diabetes, chronic inflammatory conditions, or malignancy. The patient enrollment process is illustrated in Figure 1.

### Participant selection criteria

#### Inclusion criteria

1. Observation group: Diagnosis of AMI according to the Fourth Universal Definition of Myocardial Infarction confirmed by coronary angiography
2. Control group: Healthy status confirmed by comprehensive physical examination
3. Complete clinical data, including medical records, physical examination results, and laboratory investigations

#### Exclusion criteria

1. Severe hepatic or renal insufficiency, defined as Child-Pugh class C or an estimated glomerular filtration rate <30 mL/min/1.73m^2^
2. Active malignancy
3. Other serious cardiovascular or cerebrovascular diseases
4. Hematological disorders
5. Autoimmune or systemic inflammatory diseases
6. Major surgery within the preceding 3 months

### Sample size calculation

The sample size was calculated based on detecting a clinically meaningful difference in biomarker levels between prognostic groups. Based on preliminary data and prior literature (18, 19), we estimated that patients with poor prognosis would have biomarker levels approximately 30% higher than those with good prognosis, with an expected standard deviation of 40% of the mean. Using a two-sided *α* of 0.05, power of 80%, and an anticipated MACE rate of approximately 20–25%, we determined that a minimum of 150 patients would be required. To account for potential data incompleteness, we aimed to enroll at least 170 patients. This sample size would also provide adequate statistical power for logistic regression analysis with up to 4 predictor variables, following the rule of at least 10 events per variable.

### Clinical follow-up protocol

Patients underwent systematic follow-up for 12 months post-PCI through structured telephone interviews conducted by trained research nurses blinded to baseline biomarker levels. For patients reporting potential events, medical records were obtained from treating institutions for event adjudication.

### Percutaneous coronary intervention protocol

All interventional procedures were performed by experienced operators with >100 PCI procedures annually following standard institutional protocols. Vascular access was obtained through either the radial or femoral artery, based on operator preference and patient anatomy. After local anesthesia and sterile preparation with iodophor, arterial puncture was performed using the modified Seldinger technique.

Under fluoroscopic guidance, guidewires were advanced to the coronary ostium, followed by selective coronary angiography using contrast medium to identify culprit lesions. For lesions with significant stenosis, balloon angioplasty was performed to predilate the stenotic segment. Subsequently, drug-eluting stents were deployed and post-dilated when necessary to achieve optimal apposition. Procedural success was defined as residual stenosis of <20% with TIMI flow grade 3. Following stent implantation, all equipment was withdrawn, and hemostasis was achieved using either manual compression or closure devices, according to the institutional protocol.

All patients received dual antiplatelet therapy with aspirin (100 mg daily), clopidogrel (75 mg daily), or ticagrelor (90 mg twice daily), which was initiated before the procedure and continued for at least 12 months. Additional medical therapies included statins, beta-blockers, angiotensin-converting enzyme inhibitors, or angiotensin receptor blockers, as clinically indicated.

### Baseline data collection

Comprehensive baseline characteristics were systematically collected from medical records, including demographic variables (age, sex, body mass index), cardiovascular risk factors (smoking history, alcohol consumption, hypertension), and clinical severity assessed using the Killip classification at presentation. Hypertension was defined as a systolic blood pressure ≥140 mmHg, diastolic blood pressure ≥90 mmHg, or current use of antihypertensive medications. A smoking history was defined as current smoking or cessation in the past year.

### Laboratory biomarker analysis

Venous blood samples (3 mL) were obtained from the antecubital vein using a standard venipuncture technique with minimal tourniquet application to avoid hemolysis. Samples were collected within 24 h of admission and again at 48–72 h post-PCI in serum separator tubes. After clotting at room temperature for 30 min, samples were centrifuged at 3000 rpm for 10 min at 4 °C. The separated serum was immediately processed or stored at −80 °C until batch analysis.

Serum hs-CRP concentrations were quantified using immunoturbidimetric assays (reported in mg/L) with intra-assay and inter-assay coefficients of variation of <5 and <8%, respectively. IL-18 concentrations were measured using enzyme-linked immunosorbent assay (reported in pg./mL) with similar precision. Vascular endothelial growth factor levels were determined using an enzyme-linked immunosorbent assay (Human VEGF Quantikine ELISA Kit, R&D Systems; reported in pg./mL) with a detection range of 31.2–2000 pg./mL. All assays were performed in duplicate by laboratory personnel who were blinded to clinical outcomes.

### Statistical analysis

Statistical analyses were performed using SPSS software (version 24.0; IBM Corporation, Armonk, NY, USA). Continuous variables are expressed as mean ± standard deviation and were compared using an independent samples *t*-test after confirming normal distribution with the Kolmogorov–Smirnov test. Categorical variables are presented as frequencies and percentages, and comparisons were performed using the chi-square test or Fisher’s exact test when the expected cell counts were <5.

Univariable logistic regression analysis was first performed to assess the association between each candidate predictor variable and poor prognosis. Variables with *p* < 0.10 in univariable analysis were considered for inclusion in the multivariable model. Given that traditional clinical characteristics showed no significant associations with outcome in our cohort, the final multivariable logistic regression model included only the three biomarkers (IL-18, hs-CRP, and VEGF). Results were reported as odds ratios (ORs) with 95% confidence intervals (CIs).

Receiver operating characteristic (ROC) curves were constructed to assess the discriminatory capacity of individual biomarkers for predicting adverse outcomes, with area under the curve (AUC) calculations and optimal cutoff values determined using Youden’s index. Pairwise comparisons of AUC values between biomarkers were performed using the DeLong test to evaluate whether discriminatory performance differed significantly among the three markers. Statistical significance was defined as *p* < 0.05 for all analyses.

## Results

### Study population

A total of 203 consecutive patients with AMI who underwent PCI during the study period were screened. After applying exclusion criteria, 170 patients were included in the final analysis (Figure 1). For comparison, 100 healthy individuals were enrolled as controls. The observation group comprised 97 males and 73 females with a mean age of 62.53 ± 9.97 years (range: 40–69 years). The control group included 95 males and 75 females with a mean age of 62.48 ± 9.91 years (range: 41–70 years). During the 12-month follow-up, 40 patients (23.5%) experienced MACEs and were classified into the poor prognosis group, while 130 patients (76.5%) remained event-free and constituted the good prognosis group.

### Biomarker profiles in AMI versus healthy controls

**Figure 2:**
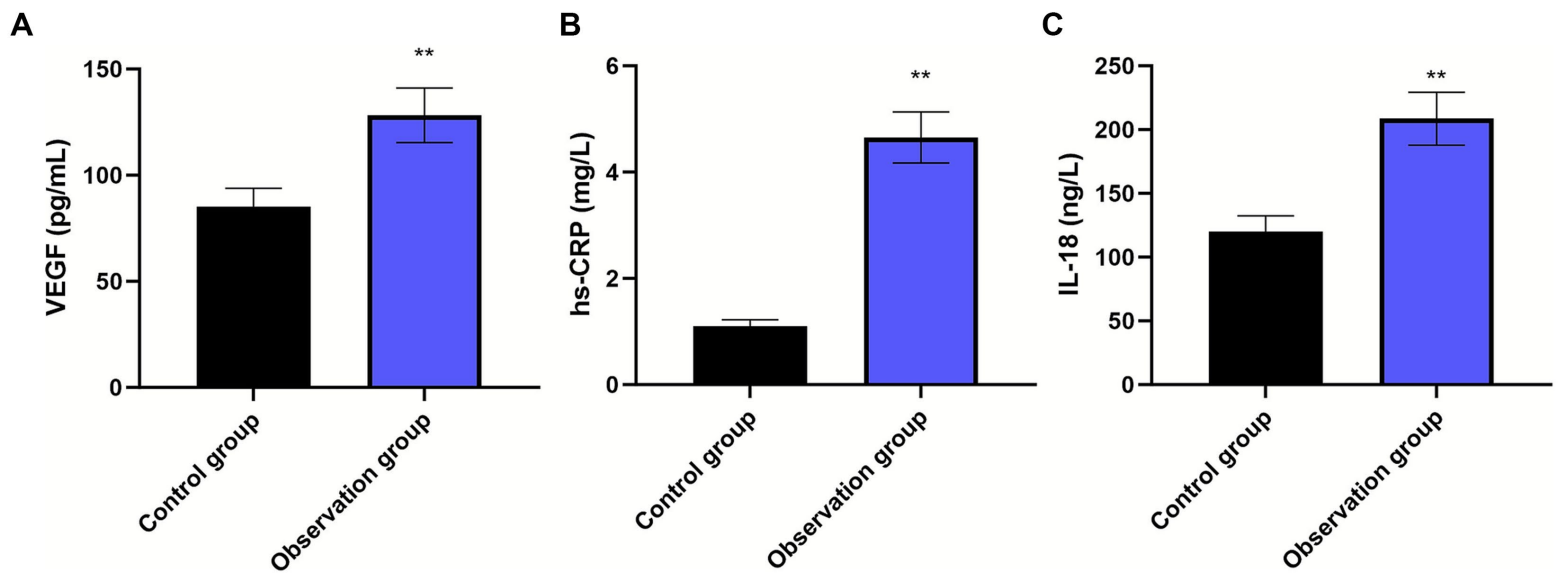
Comparison of serum biomarker levels between AMI patients and healthy controls. Bar graphs showing serum concentrations of **(A)** IL-18 (pg/mL), **(B)** hs-CRP (mg/L), and **(C)** VEGF (pg/mL) in the control group (*n* = 100) and observation group (AMI patients, *n* = 170). Data are presented as mean ± standard deviation (error bars represent SD). Groups were compared using independent samples *t*-test after confirming normal distribution with the Kolmogorov–Smirnov test. **p < 0.01 for all comparisons between groups.

Patients with AMI demonstrated substantially elevated serum concentrations of all three biomarkers compared to healthy controls (Figure 2; all *p* < 0.01). These findings confirm the activation of both inflammatory and angiogenic pathways in the acute phase of AMI and support the biological plausibility of these biomarkers as potential prognostic indicators.

### Temporal changes in biomarker levels following PCI

**Figure 3:**
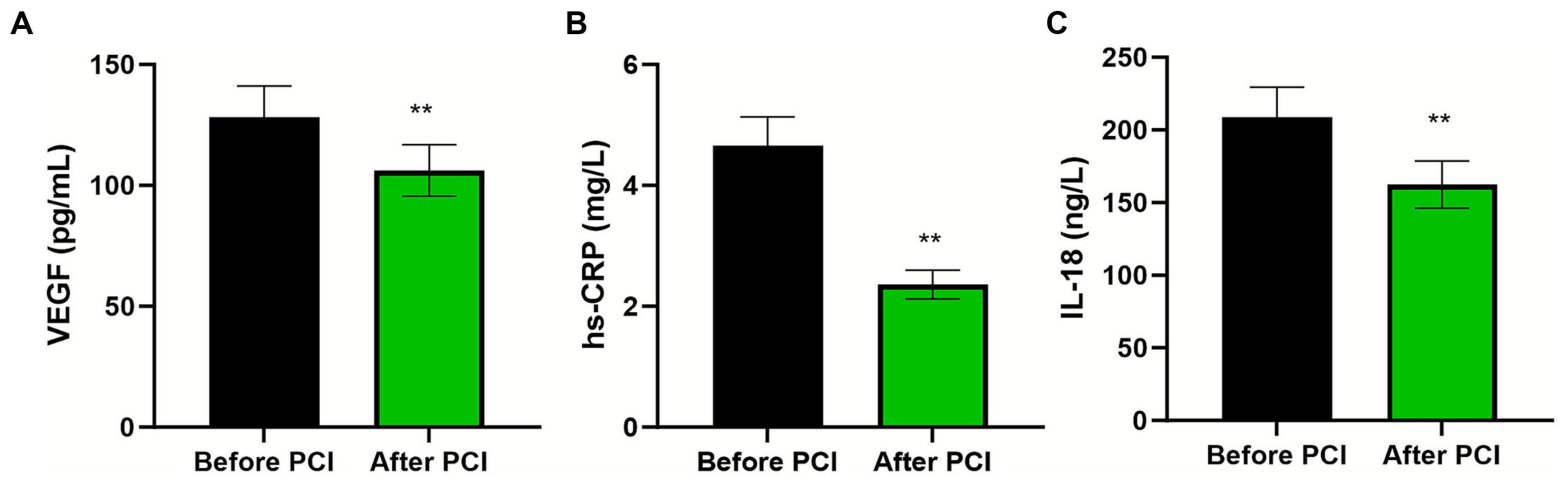
Temporal changes in biomarker levels following percutaneous coronary intervention. Paired comparison of serum **(A)** IL-18 (pg/mL), **(B)** hs-CRP (mg/L), and **(C)** VEGF (pg/mL) levels in AMI patients (*n* = 170) before and after PCI. Data are presented as mean ± standard deviation (error bars represent SD). Pre- versus post-PCI comparisons were performed using paired samples *t*-test. **p < 0.01 for all comparisons.

Post-procedural assessment revealed significant reductions in all measured parameters compared with pre-PCI values (Figure 3; all *p* < 0.01). The reduction in IL-18 and hs-CRP levels indicates attenuation of the systemic inflammatory response following successful revascularization. Similarly, the decline in VEGF concentrations reflects decreased hypoxia-driven angiogenic signaling following restoration of coronary blood flow and myocardial perfusion.

### Prognostic stratification based on pre-procedural biomarkers

**Figure 4:**
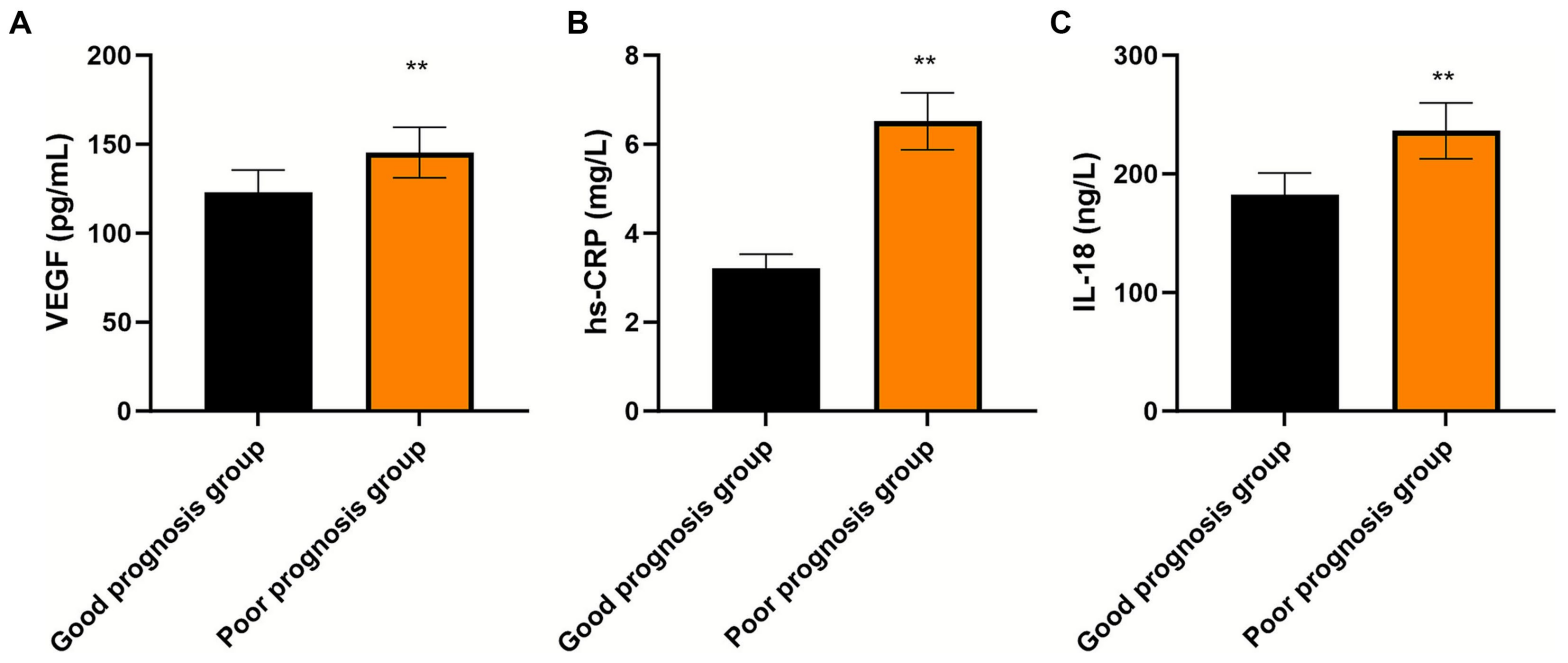
Pre-procedural biomarker levels stratified by clinical outcome. Comparison of baseline serum **(A)** IL-18 (pg/mL), **(B)** hs-CRP (mg/L), and **(C)** VEGF (pg/mL) concentrations between AMI patients with good prognosis (*n* = 130, no MACEs during 12-month follow-up) and poor prognosis (*n* = 40, experienced MACEs). Samples were obtained before PCI. Data are presented as mean ± standard deviation (error bars represent SD). Groups were compared using independent samples *t*-test. **p < 0.01 for all comparisons between groups.

Patients who subsequently experienced MACEs demonstrated significantly higher pre-procedural concentrations of all three biomarkers compared to those who remained event-free (Figure 4; all *p* < 0.01). This finding suggests that greater inflammatory activation and more pronounced ischemia-driven angiogenic responses at baseline identify patients at higher risk for adverse outcomes despite successful revascularization.

### Clinical characteristics and outcome groups

Baseline demographic and clinical characteristics showed remarkable homogeneity between patients with favorable and unfavorable outcomes (Table 1). No significant differences were observed in the distribution of traditional cardiovascular risk factors between prognostic groups, including sex, age, BMI, smoking history, alcohol consumption, hypertension, or Killip classification (all *p* > 0.05). This finding underscores the potential incremental value of biomarker assessment for risk stratification beyond traditional clinical parameters.

**Table 1:** Baseline characteristics of patients stratified by clinical outcome.

Characteristics	Poor prognosis group (*n* = 40)	Good prognosis group (*n* = 130)	Test statistic	*p* value
Demographics				
Male sex, *n* (%)	23 (57.50)	74 (56.92)	χ^2^ = 0.004	0.948
Age (years), mean ± SD	62.62 ± 10.06	62.53 ± 9.98	*t* = 0.051	0.959
BMI (kg/m^2^), mean ± SD	23.21 ± 2.35	23.18 ± 2.32	*t* = 0.073	0.942
Cardiovascular risk factors				
Current/recent smoking, *n* (%)	15 (37.50)	50 (38.46)	χ^2^ = 0.011	0.912
Alcohol consumption, *n* (%)	16 (40.00)	53 (40.77)	χ^2^ = 0.007	0.931
Hypertension, *n* (%)	11 (27.50)	42 (32.31)	χ^2^ = 0.329	0.565
Clinical presentation				
Killip classification, *n* (%)			χ^2^ = 1.141	0.767
Class I	21 (52.50)	60 (46.16)		
Class II	15 (37.50)	50 (38.46)		
Class III	3 (7.50)	12 (9.23)		
Class IV	1 (2.50)	8 (6.15)		

SD, standard deviation; BMI, body mass index.

### Univariable and multivariable predictors of adverse outcomes

Univariable logistic regression analysis was performed to identify potential predictors of poor prognosis (Table 2). Traditional clinical variables including age, sex, BMI, smoking history, alcohol consumption, hypertension, and Killip classification were not significantly associated with adverse outcomes (all *p* > 0.10). In contrast, all three biomarkers showed significant univariable associations with poor prognosis: IL-18 (OR = 28.142, 95% CI: 1.524–519.615, *p* = 0.025), hs-CRP (OR = 2.456, 95% CI: 1.142–5.281, *p* = 0.021), and VEGF (OR = 1.758, 95% CI: 1.362–2.269, *p* < 0.001).

**Table 2:** Univariable logistic regression analysis for prediction of poor prognosis.

Variable	OR	95% CI	*p* value
Demographics			
Male sex	1.025	0.497–2.115	0.948
Age (per year)	1.001	0.966–1.037	0.959
BMI (per kg/m^2^)	1.006	0.871–1.162	0.942
Cardiovascular risk factors			
Current/recent smoking	0.958	0.456–2.013	0.912
Alcohol consumption	0.969	0.469–2.002	0.931
Hypertension	0.795	0.366–1.729	0.565
Clinical presentation			
Killip class II vs. I	0.857	0.394–1.864	0.698
Killip class III vs. I	0.714	0.183–2.788	0.628
Killip class IV vs. I	0.357	0.042–3.036	0.346
Biomarkers			
IL-18 (per 1 pg./mL)	28.142	1.524–519.615	0.025
hs-CRP (per 1 mg/L)	2.456	1.142–5.281	0.021
VEGF (per 1 pg./mL)	1.758	1.362–2.269	<0.001

OR, odds ratio; CI, confidence interval; BMI, body mass index; IL-18, interleukin-18; hs-CRP, high-sensitivity C-reactive protein; VEGF, vascular endothelial growth factor.

Variables with *p* < 0.10 were considered for multivariable analysis. Only the three biomarkers met this criterion.

Multivariable logistic regression analysis confirmed that all three biomarkers remained independent predictors of poor prognosis after mutual adjustment (Table 3). IL-18 showed the strongest association (OR = 26.075 per pg./mL increase, 95% CI: 1.376–494.285, *p* < 0.001), followed by hs-CRP (OR = 2.284 per mg/L increase, 95% CI: 1.023–5.099, p < 0.001) and VEGF (OR = 1.643 per pg./mL increase, 95% CI: 1.251–2.158, p < 0.001).

**Table 3:** Multivariable logistic regression analysis for prediction of poor prognosis.

Biomarker	*β* coefficient	SE	Wald χ^2^	OR	95% CI	*p* value
IL-18 (per 1 pg./mL increase)	3.262	1.501	4.792	26.075	1.376–494.285	<0.001
hs-CRP (per 1 mg/L increase)	0.752	0.410	6.183	2.284	1.023–5.099	<0.001
VEGF (per 1 pg./mL increase)	0.496	0.139	13.002	1.643	1.251–2.158	<0.001

SE, standard error; OR, odds ratio; CI, confidence interval; IL-18, interleukin-18; hs-CRP, high-sensitivity C-reactive protein; VEGF, vascular endothelial growth factor.

Model adjusted for all three biomarkers simultaneously. Traditional clinical variables (age, sex, BMI, smoking, alcohol consumption, hypertension, Killip class) were not included in the final model as none achieved *p* < 0.10 in univariable analysis.

### Discriminatory performance of biomarkers for risk prediction

**Figure 5:**
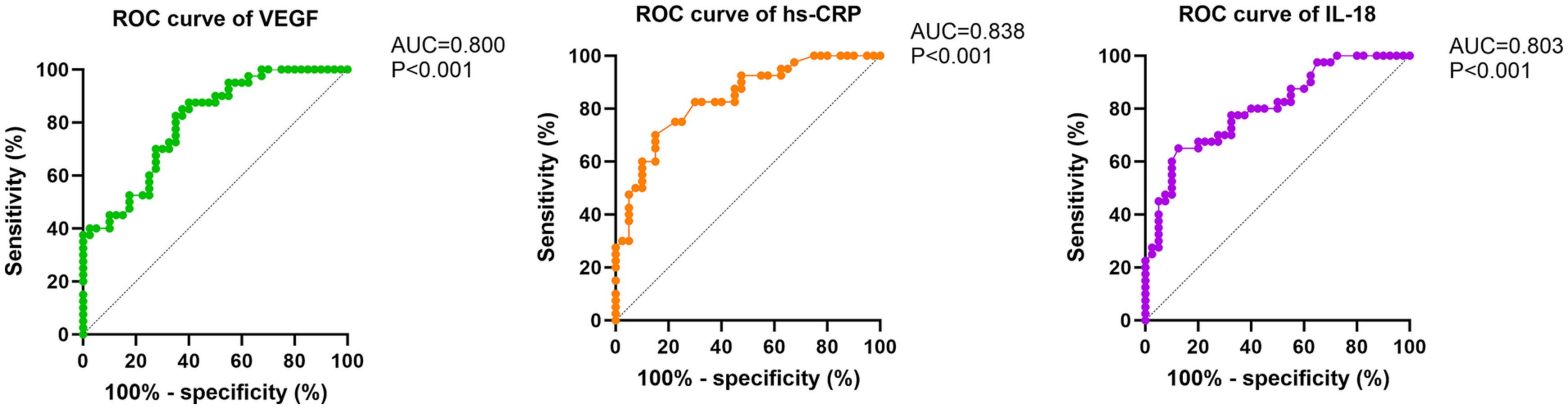
Receiver operating characteristic curves for prediction of adverse outcomes. ROC curves demonstrating the discriminatory performance of pre-procedural serum IL-18 (AUC = 0.803, 95% CI: 0.732–0.874), hs-CRP (AUC = 0.838, 95% CI: 0.774–0.902), and VEGF (AUC = 0.800, 95% CI: 0.727–0.873) levels for predicting MACEs during 12-month follow-up after PCI. All *p*-values <0.001 for AUC comparisons versus null hypothesis (AUC = 0.50). Pairwise AUC comparisons using the DeLong test showed no significant differences between biomarkers (all *p* > 0.05). Diagonal reference line indicates no discrimination.

ROC curve analysis demonstrated that all three biomarkers had robust discriminatory capacity for identifying patients at risk for adverse outcomes (Figure 5). hs-CRP showed the highest predictive accuracy (AUC = 0.838, 95% CI: 0.774–0.902), followed by IL-18 (AUC = 0.803, 95% CI: 0.732–0.874) and VEGF (AUC = 0.800, 95% CI: 0.727–0.873); all *p* < 0.001 compared to the null hypothesis of AUC = 0.50.

Pairwise comparisons of AUC values using the DeLong test revealed no statistically significant differences between biomarkers: hs-CRP vs. IL-18 (*p* = 0.421), hs-CRP vs. VEGF (*p* = 0.385), and IL-18 vs. VEGF (*p* = 0.943) (Table 4). These results indicate that while hs-CRP demonstrated numerically superior discriminatory performance, the predictive accuracy of all three biomarkers was statistically comparable.

**Table 4:** AUC comparisons using DeLong test.

Comparison	Difference in AUC	95% CI of difference	*Z* statistic	*p* value
hs-CRP vs. IL-18	0.035	−0.051 to 0.121	0.804	0.421
hs-CRP vs. VEGF	0.038	−0.048 to 0.124	0.869	0.385
IL-18 vs. VEGF	0.003	−0.085 to 0.091	0.071	0.943

DeLong test was used for pairwise comparisons of receiver operating characteristic curves. No significant differences were observed between biomarkers, indicating comparable discriminatory performance.

## Discussion

This study demonstrates that elevated preprocedural levels of IL-18, hs-CRP, and VEGF are associated with adverse outcomes in patients with AMI undergoing PCI. These biomarkers showed significant prognostic value, with AUC values exceeding 0.80, suggesting their potential utility in clinical risk stratification.

Our findings revealed significantly elevated IL-18, hs-CRP, and VEGF levels in patients with AMI compared with healthy controls, with subsequent reduction following successful PCI. These observations align with previous reports demonstrating that PCI can modulate inflammatory and angiogenic responses in AMI patients (18, 20). The postprocedural reduction in biomarker levels likely reflects decreased myocardial ischemia and attenuated inflammatory activation following revascularization.

The association between elevated pre-procedural biomarkers and poor prognosis can be explained by several mechanisms. IL-18 promotes interferon-gamma (IFN-*γ*) production, which amplifies inflammatory responses through monocyte and macrophage activation (19). Additionally, IL-18 stimulates granulocyte-macrophage colony-stimulating factor production and upregulates matrix metalloproteinase (MMP) expression in monocytes/macrophages. These MMPs, activated by mast cell-derived neutral proteinases, degrade the extracellular matrix and destabilize plaques, potentially leading to post-PCI MACEs (21).

hs-CRP contributes to atherosclerosis progression by facilitating macrophage uptake of oxidized low-density lipoproteins and foam cell formation (22). Furthermore, hs-CRP interferes with platelet function and fibrin formation, promoting a prothrombotic state that increases cardiovascular event risk despite successful revascularization (23). These mechanisms explain why elevated preprocedural hs-CRP levels predict poor outcomes, independent of procedural success.

VEGF elevation represents a compensatory response to myocardial ischemia, with hypoxia-inducible enhancer elements driving increased VEGF expression under ischemic conditions (24). While VEGF promotes therapeutic angiogenesis, persistently elevated levels may indicate severe or extensive myocardial injury that predisposes patients to adverse outcomes despite successful revascularization (15).

Logistic regression analysis confirmed that all three biomarkers independently predicted poor prognosis, with IL-18 showing the strongest association. The robust discriminatory performance of these biomarkers, particularly hs-CRP (AUC = 0.838), supports their clinical utility. These findings are consistent with those of Gao et al., who reported that IL-18 levels at admission predicted 60-day adverse events in ST-elevation AMI patients undergoing PCI (11). Similarly, previous studies have validated preprocedural CRP as a predictor of cardiovascular risk following PCI in ST-elevation AMI (9).

Notably, traditional clinical characteristics and risk factors failed to differentiate between patients with favorable and unfavorable outcomes, highlighting the incremental value of biomarker assessments. The similar distribution of Killip classification between groups suggests that these biomarkers provide prognostic information beyond clinical severity assessment. This finding aligns with emerging evidence that molecular markers may offer superior risk stratification compared with traditional clinical parameters alone.

This study has several limitations that merit consideration. The single-center retrospective design may limit the generalizability of results. The relatively small sample size, particularly in the poor prognosis group (*n* = 40), may have affected the statistical power of subgroup analyses. Additionally, we did not assess the impact of preprocedural medical therapy on baseline biomarker levels, which could influence their prognostic value. The one-year follow-up period may not capture late events, and long-term studies are warranted. Serial biomarker measurements during follow-up could provide additional insights into residual risk and treatment response. Finally, the optimal cutoff values for clinical decision making require validation in larger prospective cohorts.

Our findings suggest that pre-procedural assessment of IL-18, hs-CRP, and VEGF could enhance risk stratification in patients with AMI undergoing PCI. Patients with elevated levels may benefit from intensified medical therapy, closer monitoring, or additional interventions to mitigate risk. Future studies should evaluate whether biomarker-guided management strategies can improve clinical outcomes. Prospective multicenter studies are needed to validate these findings and to establish standardized thresholds for clinical applications.

## Conclusion

Pre-procedural elevation of serum IL-18, hs-CRP, and VEGF levels independently predicts poor outcomes in patients with AMI undergoing PCI. These biomarkers demonstrated excellent discriminatory capacity, with AUC values exceeding 0.80, supporting their potential role in clinical risk stratification. Routine assessment of these markers before PCI may help identify high-risk patients who could benefit from enhanced surveillance and targeted interventions.

## Data Availability

The original contributions presented in the study are included in the article/supplementary material, further inquiries can be directed to the corresponding author.
